# Round the Hospitals

**Published:** 1916-08-19

**Authors:** 


### ROUND THE HOSPITALS.

We understand that the members of the Poor-Law Infirmary Matrons' Association are to meet, by the invitation of Miss Barton, at Chelsea Infirmary on Saturday, 19th inst., at 3.30, when Miss Haughton and Miss Rundle will address the assembly. The subjects dealt with will no doubt include the College of Nursing and Registration. We specially direct the attention of all interested in the future of Poor-Law nursing to page 475, where information of the first importance will be found under the heading "National Poor-Law Officers' Association."

---

Why is it that many well-known Poor-Law infirmaries are so defective in their nursing arrangements as to fail to provide off-duty time-tables for the nursing staff, with the result that the majority of them seldom or never throughout the year obtain outdoor exercise daily in daylight? We have been surprised to hear it stated with authority that the hours on duty for nurses and probationers in these institutions are usually twelve, with about half an hour off for each meal, but no two or three hours off during the working day, as is usual in well-administered hospitals. Having regard to the locality in which many infirmaries are necessarily situated and to other circumstances, it is clearly not right for any Poor-Law infirmary to send its nurses out at all seasons after 8 p.m., should they want outdoor exercise. Some of the best nurses for private nursing have been trained in the best of the Poor-Law infirmaries, but it is clear on hygienic grounds that if the evils of continuous duty without adequate outdoor exercise are pursued much longer in the establishments in question, they may acquire an unenviable reputation from sickness amongst their staff, with a general loss of energy and brightness, two factors which have a special value in attendants upon the sick.

---

The matron of the Hackney Union Infirmary (Miss Griffith) has arranged that her probationers should attend lectures and classes, both those given by the medical superintendent and those by herself, in the forenoon of each day instead of in the evenings, the usual time fixed in most training schools. Miss Griffith contends that any initial trouble in the necessary rearrangement of the day's work has been amply repaid by the freshness with which her probationers have been able to come to their classes and by the progress they have made under the new system. The sisters of the Hackney Union Infirmary feel they, too, are repaid for any small inconveniences arising from the change, because the probationers seem to find the morning lectures and classes of real use and help to them in the wards. This was not invariably the case when the lectures and classes were held at the end of a long day's hard work, in which circumstances the weary pupils were not able to obtain any real benefit, or to add appreciably to their knowledge. We hope morning lectures and classes will become general in Poor-Law infirmaries.

---

It is interesting to note that Dr. Lewis Hawkes, whose wife, Mrs. Coghill Hawkes, is a qualified medical practitioner, and also a member of the Swedish Institute and Clinique, Cromwell Road, S.W., when addressing the members of the Incorporated Society recently on medical ethics, was most outspoken. He urged, for instance, that members of all societies were bound by both legal and moral obligations. Members of the Incorporated Society of Trained Masseuses were under three legal obligations—*i.e.* to work only under medical men; not to advertise; and not to sell drugs. The moral obligations of masseuses were to the medical profession, their patients, their Society, and themselves. When a masseuse took up her residence in a district, she should remain for at least six months, call upon all medical practitioners within it, never give treatment without the practitioner's instructions, and invariably be businesslike by always having a visiting-card with her name, address, qualifications, and telephone number, one or two good testimonials, and her scale of fees. Whenever possible, the masseuse should obtain from the practitioner, at a personal interview preferably, his diagnosis of the case, with written instructions as to the treatment he wishes applied.

---

In her attendance on the patient the masseuse should make it an invariable rule to avoid in conversation such topics as religion, the suffrage, politics; never suggest any other form of treatment than that she is instructed to adopt, and never to discuss with the patient the practitioner's or any other patient. A tactful avoidance of the danger points just mentioned, a pleasant manner to everybody, and a habit of dressing quietly but becomingly, were aids to success in practice. Their duty to themselves entailed something more than the mere passing of examinations, for a good foundation to successful practice meant initially much hard work, the common sense to play hard in their leisure hours and to feed wholesomely and well. A masseuse should acquire the habit of doing her hair to suit her face, of taking great care of her hands, teeth, and feet, and avoid always the habit of taking drugs, mannerisms—*i.e.*, irritating little coughs, sucking their teeth or lips, and so forth. They should give special attention to their underwear and to clothes. Dr. Hawkes would seem to have an intimate acquaintance with the way to succeed as a masseuse, but his remarks and much of his advice have a much wider application, as every experienced worker in the field of nursing will gratefully recognise. It might help Dr. Lewis Hawkes and prove instructive reading to learn the views of matrons, sisters, and trained masseuses on medical ethics as expounded by him.

For Matrons' and Sisters' Appointments see p. 480.

